# La resistencia a la insulina como factor etiológico en el síndrome del ovario poliquístico: un estudio de casos y controles

**DOI:** 10.1515/almed-2022-0050

**Published:** 2022-06-17

**Authors:** Jyoti R. Singh, Anju Jain, Nishtha Wadhwa, Tilak H.R., Ashok Kumar Ahirwar

**Affiliations:** Indian Council of Medical Research (ICMR), Delhi, India; Department of Biochemistry, Lady Hardinge Medical College, New Delhi, India; SRL Diagnostics, Gurugram, India; Bhandari Hospital and Research Center, Indore, MP, India; Assistant Professor, Department of Biochemistry, University College of Medical Sciences, New Delhi, 110095, India

**Keywords:** normoglucemia, resistencia a la insulina, síndrome del ovario poliquístico (SOP)

## Abstract

**Objectivos:**

Aunque la relación entre la resistencia a la insulina (RI) y el síndrome del ovario poliquístico (SOP) ha sido demostrada en diversos estudios, los mecanismos subyacentes de causa y efecto aún no han sido dilucidados. En los últimos años, se ha señalado que la RI podría ser un factor etiológico clave, asociado a la gravedad de los desórdenes metabólicos y reproductivos de las pacientes con SOP. El objetivo del presente estudio es determinar el papel de la RI en la etiología del SOP.

**Métodos:**

Se realizó un estudio analítico de casos y controles, en el que se incluyó a 30 pacientes normoglucémicas con SOP (definido conforme a los criterios revisados de Rotterdam 2003) con edades comprendidas entre los 15 y los 35 años. En el grupo de control se incluyó a 30 voluntarias sanas de edades similares. La glucosa en ayudas se analizó mediante espectrofotometría, mientras que la insulina en ayunas se midió mediante inmunoensayo de quimioluminiscencia. Los valores de HOMA-IR, Log HOMA-IR, QUICKI, G/I y FIRI se calcularon aplicando las fórmulas estándar.

**Resultados:**

en el grupo de casos, los parámetros antropométricos y marcadores de RI estaban elevados, mientras que los índices QUICKI y HOMA fueron inferiores, comparados con el grupo de controles (p<0,05). Las pacientes con un IMC ≥25 mostraron valores más elevados en los marcadores de RI, así como unos índices QUICKI y G/I menores que las pacientes con un IMC <25, y que los controles con un IMC similar. No se observaron diferencias significativas en los marcadores de RI entre las pacientes con alta y baja obesidad central.

**Conclusiones:**

En las pacientes con SOP obesas y normoglucémicas, la elevación de los marcadores de RI no se puede atribuir únicamente a la obesidad o a la obesidad central. La presencia temprana de RI en pacientes con un diagnóstico reciente de SOP, incluso antes de desarrollar hiperglucemia e hiperinsulinemia, señala a la RI como un factor causal en el desarrollo del SOP.

## Introducción

El síndrome del ovario poliquístico (SOP) es el trastorno endocrino más común entre las mujeres en edad reproductiva, teniendo diversas implicaciones y estando presente en hasta el 18% de este grupo de población [1, 2]. El SOP se caracteriza por hiperandrogenismo, disfunción ovárica y/o una morfología micro-poliquística del ovario. Sin embargo, se pueden observar otras alteraciones no incluidas entre las características de diagnóstico. De este modo, las mujeres con SOP presentan un mayor grado de resistencia a la insulina (RI) [1, 3], definida como un estado en el que se requiere una cantidad de insulina superior a la normal para inducir una respuesta [4].

Varios estudios demuestran la existencia de resistencia a la insulina e hiperinsulinemia compensatoria en aproximadamente el 80% de las mujeres obesas con SOP, y en el 30–40% de las mujeres delgadas [5]. Aparentemente, el mecanismo de RI es una anomalía de unión en la transducción de señales mediada por receptores de la insulina [6].

Aunque la relación entre la resistencia a la insulina (RI) y el síndrome del ovario poliquístico (SOP) ha sido demostrada en diversos estudios, los mecanismos subyacentes de causa y efecto aún no han sido dilucidados [1]. Así, se desconocen el origen y los mecanismos de la resistencia a la insulina en el SOP [7].

En los últimos años, se ha señalado que la RI podría ser un factor etiológico clave, asociado a la gravedad de los desórdenes metabólicos y reproductivos de las pacientes con SOP, cuyos síntomas clínicos empeoran con la obesidad, en parte por la estimulación de la esteroidogénesis y la producción de andrógenos [8, 9].

El SOP es un trastorno endocrino difícil de manejar [9]. Las modificaciones en el estilo de vida son la piedra angular en el tratamiento del SOP. Se ha demostrado que perder peso y realizar ejercicio físico, así como reducir la obesidad central y mejorar la RI, mejoran los problemas menstruales y la infertilidad en las mujeres obesas con SOP [10]. En las pacientes con sobrepeso y obesidad, la pérdida de peso reduce los niveles séricos de insulina y andrógenos, disminuyendo así el riesgo de desarrollar diabetes tipo 2 [11]. Así, determinar cuantitativamente la sensibilidad a la insulina podría ayudar a identificar a las pacientes con mayor riesgo de desarrollar secuelas metabólicas como la diabetes, así como a aquellas que podrían responder mejor al tratamiento con agentes sensibilizadores de insulina [4]. El objetivo del presente estudio es determinar el papel de la RI en la etiología del SOP.

## Materiales y métodos

Se realizó un estudio analítico de casos y controles, en el que se incluyó a 30 pacientes normoglucémicas (glucosa en sangre < 100) con SOP (establecido conforme a los criterios revisados de Rotterdam 2003) [12], con edades comprendidas entre los 15 y los 35 años. En el grupo de control se incluyó a 30 voluntarias sanas de edades similares a la de los casos. El estudio se llevó a cabo en la clínica de ginecología y adolescencia Lady Hardinge Medical College and associated Hospitals entre noviembre de 2012 y marzo de 2014, tras obtener el consentimiento informado por escrito en formato bilingüe de las participantes. Este estudio ha sido aprobado por el Comité Ético Institucional y realizado según los principios de la Declaración de Helsinki. Se excluyó a las mujeres con patologías relacionadas, como la diabetes mellitus, la hiperprolactinemia, los tumores secretores de andrógenos, la hiperplasia suprarrenal y la osteomalacia.

Se sometió a las pacientes a un examen físico, donde se midieron parámetros antropométricos, esto es, altura en cm, peso en kg, perímetro abdominal en cm, perímetro de cadera en cm, índice cintura-cadera (ICC), calculado aplicando la fórmula Perímetro de la cintura (cm)/perímetro de la cadera (cm); y el índice de masa corporal (IMC), calculado con la fórmula Peso (kg)/(altura en m)^2^.

A primera hora de la mañana, en el 2° y 5° día del ciclo menstrual, se tomaron muestras de sangre venosa en ayunas, con el fin de analizar la glucosa en ayunas mediante espectrofotometría y la insulina mediante inmunoensayo de quimioluminiscencia.

– HOMA-IR (modelo hosmeostático para evaluar la resistencia a la insulina), calculado con la siguiente fórmula: HOMA-IR=Glucosa (mg/dl)×Insulina (µIU/mL)/405 [13].
– Log HOMA-IR, calculado con el algoritmo de HOMA-IR [13].
– QUICKI (índice cuantitativo de sensibilidad a la insulina), calculado con la fórmula 1/log Insulina (µIU/mL)+log glucosa (mg/dL) [13].
– Índice glucosa/Insulina (índice G/I) [13].
– FIRI (índice de resistencia a la insulina en ayunas), calculado con la fórmula (Glucosa en ayunas x insulina en ayunas)/25 [13].

### Análisis estadístico

Los datos obtenidos en el estudio se analizaron con Microsoft Excel 365 y SPSS versión 20. Los resultados se expresan en medias ± DE (desviación estándar). La normalidad de la distribución se comprobó con la prueba de Shapiro-Wilk. Las diferencias entre grupos se analizaron, dependiendo de la normalidad, bien mediante la prueba t de Student o la prueba U de Mann-Whitney. Un valor bilateral p<0,05 se consideró estadísticamente significativo, mientras que un valor p<0,001 se consideró estadísticamente muy significativo.

## Resultados

En la Tabla 1 se comparan los valores antropométricos y de los marcadores de IR de los casos y controles. El grupo de casos mostró unos valores antropométricos y unos marcadores de IR significativamente superiores, siendo los índices QUICKI y G/I inferiores que en los controles.

**Tabla 1:** Comparación de los parámetros antropométricos y marcadores de RI entre pacientes normoglucémicas y controles.

Parámetros antropométricos Y marcadores DE RI	Casos (n=30)	Controles (n, X%)	Valor p
IMC	26,25 ± 5,32	23,36 ± 3,29	0*,*015^a^
ICC	0,861 ± 0,061	0,798 ± 0,060	<0*,*001^b^
Insulina en ayunas	11,55 ± 10,80	5,63 ± 2,55	0*,*006^a^
HOMA- IR	2,47 ± 2,40	1,17 ± 0,53	0*,*007^a^
Log HOMA-IR	0,26 ± 0,33	0,03 ± 0,19	0*,*002^a^
QUICKI	3,12 ± 0,42	3,46 ± 0,50	0*,*006^a^
Índice glucosa/insulina	11,94 ± 7,19	18,31 ± 8,73	0*,*003^a^
FIRI (índice de resistencia a la insulina en ayunas)	39,94 ± 38,89	18,97 ± 8,72	<7*,*0%^a^

^a^Un valor p<0,05 se consideró significativo. ^b^Un valor p<0,001 se consideró altamente significativo.

La resistencia a la insulina fue significativamente superior en el grupo de casos, pese a ser normoglucémicas, frente a los controles. No está claro si la RI en las pacientes con SOP es a causa de su elevado IMC o elevada obesidad central o debido al propio SOP (Tabla 2).

**Tabla 2:** Comparación de los marcadores de RI en pacientes normoglucémicas obesas y delgadas.

Marcadores DE RI	IMC ≥25 (n=16)	IMC <25 (n=14)	Valor p
Insulina en ayunas	15,23 ± 13,25	7,35 ± 4,73	0*,*039^a^
HOMA- IR	3,27 ± 0,2,93	1,54 ± 1,10	0*,*041^a^
Log HOMA-IR	0,385 ± 0,33	0,108 ± 0,26	0*,*018^a^
QUICKI	2,96 ± 0,31	3,29 ± 0,47	0*,*038^a^
Índice glucosa/insulina	9,33 ± 6,07	14,92 ± 7,41	0*,*034^a^
FIRI (índice de resistencia a la insulina en ayunas)	53,01 ± 47,49	24,97 ± 17,86	0*,*041^a^

^a^Un valor p<0,05 se consideró significativo.

Para averiguarlo, se dividió a 30 pacientes en dos grupos según su IMC, esto es, pacientes obesas (IMC ≥25) y delgadas (IMC<25) [14], y se compararon los valores de los marcadores de RI. Tal como se muestra en la Tabla 3, las mujeres con sobrepeso y con obesidad exhibieron valores superiores en los marcadores de RI, e índices QUICKI y G/I inferiores, frente a las mujeres delgadas, siendo las diferencias estadísticamente significativas. Esto sugiere que, en las pacientes con SOP, la RI está estrechamente correlacionada con un IMC elevado. Para determinar si la obesidad está relacionada de forma independiente o no con la RI, se realizó una comparación entre las pacientes obesas normoglucémicas y los controles con un IMC similar. Tal como indica la Tabla 3, las pacientes con un IMC ≥25 mostraron valores significativamente superiores en los marcadores de RI, así como índices QUICKI y G/I inferiores que el grupo de control, lo que indica que la RI elevada en las pacientes obesas no puede ser atribuida únicamente a la obesidad. Además, para evaluar la obesidad central, se dividió a las pacientes según si presentaban un ICC elevado (≥0,85) o bajo (<0,85), y se analizaron los valores de los marcadores de RI. Tal como indica la Tabla 4, no se observaron diferencias significativas en los marcadores de RI entre las pacientes con alta y baja obesidad central, lo que indica que la obesidad central podría no estar relacionada con el desarrollo de la RI en el SOP.

**Tabla 3:** Comparación de los marcadores de RI entre pacientes obesas normoglucémicas y controles con IMC similar.

Marcadores DE RI	Casos (n=16)	Controles (n=10)	Valor p
Insulina en ayunas	15,23 ± 13,25	5,67 ± 3,56	0*,*014^a^
HOMA-IR	3,27 ± 2,93	1,21 ± 0,78	0*,*016^a^
Log HOMA-IR	0,39 ± 0,336	0,01 ± 0,24	0*,*004^a^
QUICKI	2,96 ± 0,31	3,51 ± 0,45	0*,*005^a^
Índice glucosa/insulina	9,33 ± 6,07	19,16 ± 7,92	0*,*004^a^
FIRI (índice de resistencia a la insulina en ayunas)	53,03 ± 47,49	19,64 ± 12,74	0*,*016^a^

^a^Un valor p<0,05 se consideró significativo.

**Tabla 4:** Comparación de marcadores de RI entre las pacientes con obesidad central elevada y baja.

Marcadores DE RI	ICC ≥0,85 (n=18)	ICC <0,85 (n=12)	Valor p
Insulina en ayunas	11,85 ± 7,60	11,11 ± 14,77	0*,*874
HOMA- IR	2,52 ± 1,77	2,39 ± 3,21	0*,*904
Log HOMA-IR	0,303 ± 0,30	0,19 ± 0,37	0*,*375
QUICKI	3,02 ± 0,32	3,26 ± 0,53	0*,*187
Índice glucosa/insulina	10,28 ± 6,08	14,43 ± 8,24	0*,*151
FIRI (índice de resistencia a la insulina en ayunas)	40,75 ± 28,76	38,72 ± 52,02	0*,*903

## Discusión

En el presente estudio, observamos que la RI era significativamente mayor en las pacientes con SOP, a pesar de ser normoglucémicas, que en los controles, lo que sugiere que la RI ejerce un papel relevante en la patogénesis del SOP. Un estudio similar realizado por Jing Gao et al. en población China reveló que las pacientes con SOP con tolerancia normal a la glucosa presentan mayor resistencia a la insulina que los controles [15].

En el SOP, se produce la llamada “paradoja central”, mediante la cual, a pesar de hallarse en un estado de resistencia a la insulina, el ovario sigue siendo sensible a la acción de la insulina para producir andrógenos. Existen evidencias de que las pacientes con SOP muestran un número y afinidad óptimos de receptores de la insulina en diferentes tejidos diana de la insulina, así como en los ovarios, no habiéndose detectado alteraciones estructurales o mutacionales en los receptores de la insulina [16]. Así, parece que se trata de un defecto de unión post-receptor en la vía de señalización de la insulina el que desempeña un papel importante en la etiología de la resistencia selectiva a la insulina [6, 17].

Diferentes estudios muestran que la hiperinsulinemia y la RI son los mecanismos centrales en la patogénesis del SOP [4, 10], aunque son escasos los estudios sobre la etiología del SOP [7, 8].

Al comparar la RI en las pacientes obesas y delgadas, observamos que esta era significativamente mayor en las pacientes obesas, lo que indica una fuerte correlación entre la obesidad y el grado de RI. A la luz de los resultados obtenidos, comparamos a las pacientes obesas con un grupo de control con un IMC similar, observando que la RI era significativamente mayor en el grupo de casos. La ausencia de RI en el grupo de control con IMC similar indica que la obesidad podría no ser la única responsable de la RI, lo que concuerda con los resultados obtenidos por Park et al. y Evans et al. [14, 18, 19].

Al evaluar la obesidad central, no se halló relación alguna entre esta y la RI. Esto indica que, en las pacientes con un diagnóstico reciente de SOP, la obesidad central no está relacionada con el desarrollo de RI. Park et al. y Evans et al. también afirman que la sensibilidad a la insulina está relacionada solo en parte con las diferencias en el ICC, esto es, en la obesidad central, más que en la adiposidad general [18, 19].

La presencia de RI en los casos recientes de SOP indica que la RI desempeña un papel relevante en la etiología del SOP. De este modo, desde un principio, se debería evaluar la RI en las mujeres normoglucémicas con SOP, con el fin de adoptar medidas que mejoren el metabolismo de la glucosa, tales como la pérdida de peso, el ejercicio físico regular, la dieta, y otras modificaciones en el estilo de vida que se puedan adoptar inmediatamente tras el diagnóstico [15, 20].

### Limitaciones

Una de las principales limitaciones de este estudio es el tamaño muestral. Se estableció un tamaño de muestra fácilmente alcanzable, debido al breve periodo de estudio. Únicamente se emplearon marcadores sustitutivos de RI, frente al método estándar para determinar la sensibilidad a la insulina, esto es, el método de *clamp* hiperinsulinémico. En este estudio no se determinaron los niveles de HbA_1c_. Son necesarios más estudios de seguimiento para evaluar la causa, mecanismo y base molecular de la RI en estas pacientes.

## Conclusiones

Los resultados de este estudio indican que las mujeres normoglucémicas con SOP presentan valores significativamente elevados en los marcadores de RI, frente a los controles con edad e IMC similares. La RI en estas pacientes no se puede atribuir únicamente a la obesidad o a la obesidad central. Se trata de pacientes normoglucémicas recientemente diagnosticadas, que presentan RI en las primeras fases de la enfermedad, incluso antes de que se produzcan alteraciones de la glucosa en sangre y la insulina. Estos hallazgos sugieren que estas pacientes desarrollan RI mucho antes de desarrollar el SOP, lo que indica que la RI es un factor causante en el desarrollo del SOP.
